# Discovery of HB-EGF binding peptides and their functional characterization in ovarian cancer cell lines

**DOI:** 10.1038/s41420-019-0163-9

**Published:** 2019-03-25

**Authors:** Yanting Shen, Lingling Ruan, Caixia Lian, Ruyan Li, Zhigang Tu, Hanqing Liu

**Affiliations:** 1 School of Pharmacy, Jiangsu University, Zhenjiang, 212013 Jiangsu China; 2 Institute of Life Sciences, Jiangsu University, Zhenjiang, 212013 Jiangsu China

## Abstract

Ovarian cancer is one of the most frequent causes of cancer death among all gynecologic cancers. Though standard therapy often results in temporary clinical remission, most patients suffer from recurrence and metastasis of ovarian cancer, which highlights the need for developing new therapeutic agents targeting specific molecules. Previous studies have demonstrated that the native ligand of epidermal growth factor receptor (EGFR) and ErbB4, heparin-binding EGF-like growth factor (HB-EGF), plays a critical role in the progression of ovarian cancer and is associated with prognosis of ovarian cancer. In the current study, we tried to develop a peptide-based treatment for ovarian cancer by targeting HB-EGF. After the functions of HB-EGF in promoting migration and invasion of SKOV3 and HO-8910 cells were confirmed, phage display was used to discover peptides binding to HB-EGF. Two peptides, no. 7 and no. 29 were found mildly binding to HB-EGF. Then the effects of these peptides on HB-EGF functions were examined and both peptides no. 7 and no. 29 were found indeed inhibiting the functions of HB-EGF in promoting migration and invasion of SKOV3 and HO-8910 cells in vitro. Further mechanism investigation showed that peptides no. 7 and no. 29 inhibited HB-EGF-promoted cell migration and invasion through attenuating activation of the EGFR signaling pathway manifested by decreased p-Erk1/2 and Snail levels. More importantly, peptides no. 7 and no. 29 showed strong activities in inhibiting migration of SKOV3 cells in vivo. These results provide a proof of concept method for developing novel peptide drugs to combat ovarian cancer through interfering with HB-EGF mediated signaling pathways.

## Introduction

Ovarian cancer is one of the most frequent causes of cancer death among all gynecologic cancers^1^. It has been proved that heparin-binding EGF-like growth factor (HB-EGF), an important ligand of epidermal growth factor receptor (EGFR), participates in tumorigenesis and development of ovarian cancer^2^ and also is a useful biomarker for cancer prognosis^3,4^. Among EGFR ligands, expression of HB-EGF is the highest in various ovarian cancer cell lines^5^ and in malignant ovarian cancer patients^6^. Initially synthesized as a type I transmembrane protein, proHB-EGF can be cleaved by a disintegrin and metalloprotease (ADAM) or a matrix metalloproteinase (MMP) through a process known as “ectodomain shedding” to release soluble HB-EGF (sHB-EGF, the mature form) and C-terminus of proHB-EGF (HB-EGF-C)^7–9^. Studies of knock-in mice expressing an uncleavable mutant form of HB-EGF indicated that the major functions of HB-EGF were mediated by sHB-EGF in vivo^10^. In addition, sHB-EGF was reported to significantly contribute to cancer progression through promoting survival, adhesion, invasion, and angiogenesis of cancer cells^11,12^.

As a native ligand of EGFR and ErbB4, sHB-EGF exerts its activities by binding to its receptors^6,10^. Further studies showed that HB-EGF is a promising drug target for ovarian cancer therapeutics^13–17^. So far, several anti-HB-EGF strategies have been developed for cancer treatment^18–20^. Tumor formation of ovarian cancer cells could be blocked by RNA interference targeting HB-EGF or by CRM197, a specific HB-EGF inhibitor^6^. A series of anti-HB-EGF monoclonal antibodies have been generated and they exhibited antitumor effects^16,21^.

Peptide drugs stand as a novel kind of medicines in the current pharmaceutical development. Peptide drugs possess several advantages over other macromolecules drugs such as antibodies and proteins, including lower immunogenicity and better bioavailability. Till now, over 50 peptide-based products have been approved for clinical use^22^.

Invented by George P. Smith, phage display is a practical technology to discover brand new bioactive peptides and antibodies. Gregory P. Winter and others further developed this technology. A half share of the 2018 Nobel Prize in chemistry was awarded to Smith and Winter for their great contribution to phage display technology.

Inspired by these previous studies, we sought to discover bioactive peptides inhibiting functions of sHB-EGF. The functions of sHB-EGF on ovarian cancer cell lines SKOV3 and HO-8910 were first confirmed. Peptides which can bind sHB-EGF were then screened out using phage display. After the sHB-EGF binding peptides were obtained, we tested the effects of peptides on functions of sHB-EGF and tried to dissect the underlying mechanism. The results of this study will provide a proof-of-concept method for developing novel peptide drugs to combat ovarian cancer through interfering with HB-EGF-mediated signaling pathways.

## Results

### Overexpression and purification of recombinant human sHB-EGF

**Fig. 1:**
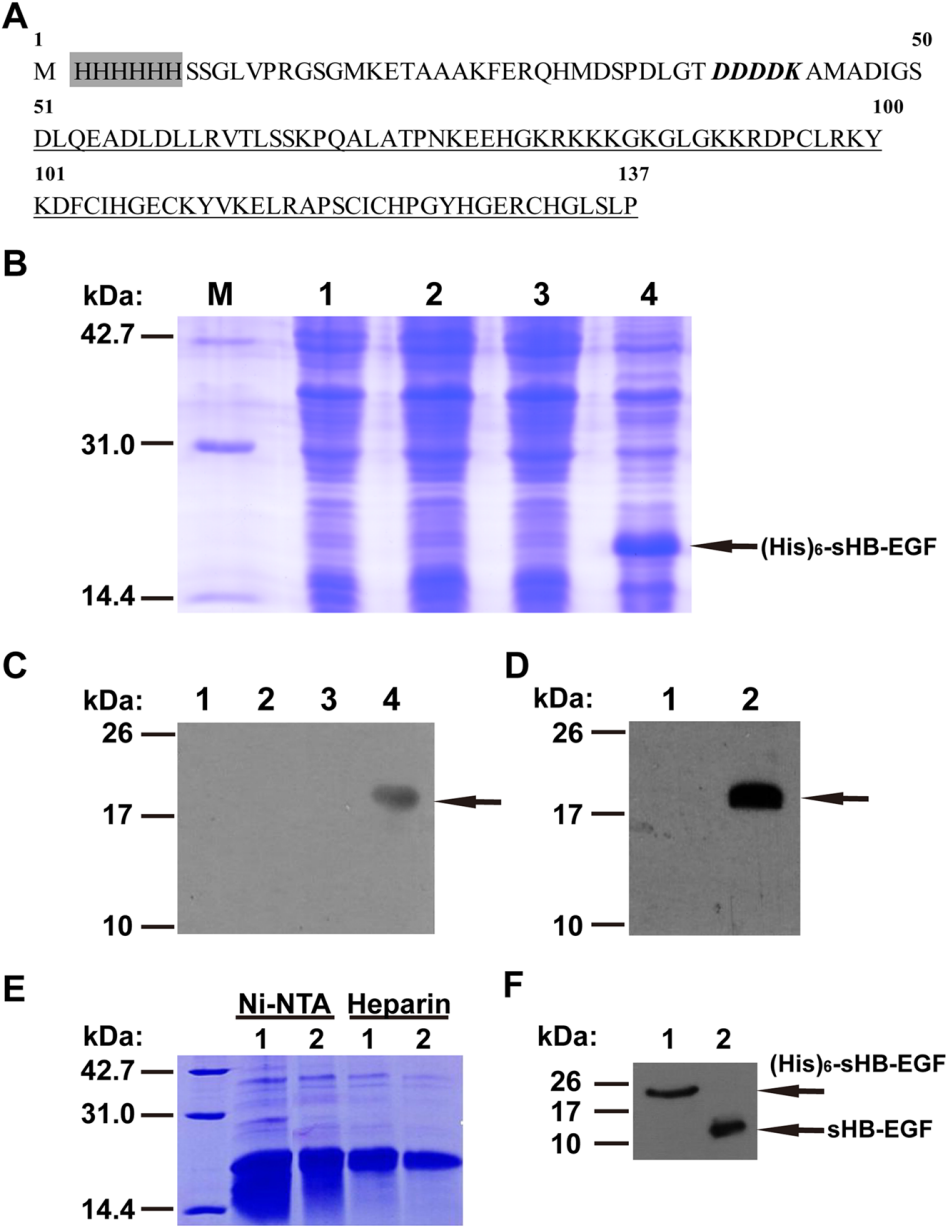
Production and purification of sHB-EGF. **a** Amino acid sequence of recombinant protein (His)_6_-sHB-EGF. The shadow area remarks His-tag (6 × His). The bold italic part indicates enterokinase cutting site. The underlined part represents human sHB-EGF (63–149 AA). **b** Verification of the expression of recombinant protein through 15% SDS-PAGE. M: protein marker, Lane 1: the lysate of BL21; Lane 2: BL21 containing pET-30a; Lane 3: BL21 containing pET-30a-His-HB-EGF without IPTG induction; Lane 4: BL21 containing pET-30a-His-HB-EGF with IPTG induction. **c** Verification of the expression of recombinant protein by western blot. Lanes 1–4 are identical to those in **b**. The antibody used here is anti-His antibody. **d** Verification of the expression of recombinant protein by western blot. Lane 1: the lysate of BL21 containing pET-30a-His-HB-EGF without IPTG induction; Lane 2: above BL21 with IPTG induction. The antibody used was anti-HB-EGF antibody. **e** SDS-PAGE after Ni-NTA and heparin affinity chromatography. Lanes 1 and 2: two most concentrated elution fractions from each affinity chromatography. **f** Detection of protein before and after enterokinase digestion by western blot. Lane 1: before enterokinase digestion. Lane 2: after enterokinase digestion. The antibody used was anti-HB-EGF antibody

Since sHB-EGF exerts the major functions of HB-EGF, we therefore focused our research on sHB-EGF. As the first step, a pET-30a-His-sHB-EGF expression plasmid was constructed to overexpress recombinant human sHB-EGF in BL21 (DE3) cells. As shown in Fig. 1a, the first 50 amino acids are translated from the vector (pET-30a) where a His-tag and an enterokinase site (DDDK) exist, and the sequence covering from 51st to 137th amino acid represents human sHB-EGF (63–149 AA). When BL21 cells containing pET-30a-His-sHB-EGF plasmid were induced with 0.8 mM of IPTG, they expressed a specific and dense protein band compared to empty BL21, BL21 containing pET-30a vector or BL21 cells containing pET-30a-His-sHB-EGF plasmid but without IPTG induction (Fig. 1b). In addition, western blot with anti-His or anti-HB-EGF antibody was used to confirm the existence of (His)_6_-sHB-EGF in BL21 cells containing pET-30a-His-sHB-EGF plasmid after IPTG induction (Fig. 1c, d). Next, affinity chromatography with Ni-NTA beads and heparin sepharose (Fig. 1e) were performed sequentially, and (His)_6_-sHB-EGF with a purity over 95% (by estimating the area and density of the protein bands using NIH ImageJ software) was obtained. Furthermore, we cleaved (His)_6_-sHB-EGF protein with enterokinase and retrieved sHB-EGF with a purity of 91.1% (tested by HPLC, Fig. S1) using heparin sepharose (Fig. 1f).

### sHB-EGF treatment mildly increased the viabilities of SKOV3 and HO-8910 cells

To probe whether our purified sHB-EGF has similar activities in human ovarian cancer lines reported in previous studies^21^, we first studied the effects of HB-EGF on proliferation of SKOV3 and HO-8910 cells. It was revealed that sHB-EGF treatment only mildly increased the viabilities of SKOV3 cells (Fig. S2A). This growth-promoting effect of sHB-EGF seemed to reach a plateau when the concentration of sHB-EGF reached 0.0625 μg/ml. In addition, we found sHB-EGF exerted similar growth-promoting effect on HO-8910 cells (Fig. S2B). Based on these results, we chose 0.125 μg/ml as the concentration for sHB-EGF treatment in the following experiments as it may safely exert the function of sHB-EGF and also avoid side effects due to the treatment with an unnecessary high dosage.

### sHB-EGF significantly increased migration and invasion of SKOV3 and HO-8910 cells

**Fig. 2:**
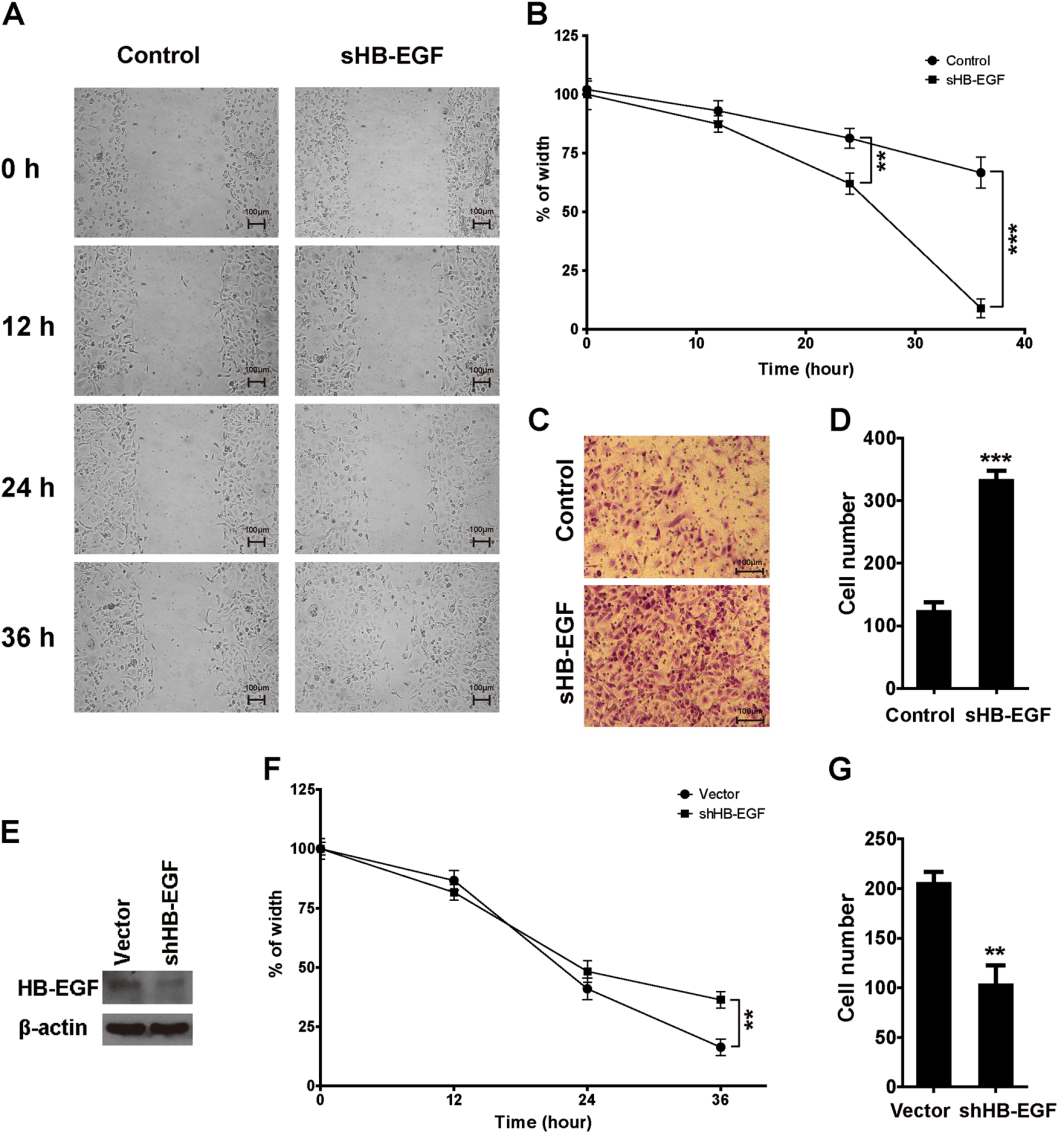
sHB-EGF promoted migration and invasion of SKOV3 cells. **a** The representative pictures of wound-healing assay. For sHB-EGF treatment, sHB-EGF was added to 0.125 μg/ml. **b** Statistical analysis of wound-healing assays. **c** The representative pictures of transwell invasion assay. **d** Statistical analysis of transwell invasion assay. **e** HB-EGF expression levels in SKOV3 cells with or without knockdown. **f**, **g** Statistical analysis of wound-healing assay and invasion assay on SKOV3 cells with or without HB-EGF knockdown. Mean ± SD, *n* = 3. **P < 0.01, ***P < 0.001; compared with the control group

After the concentration of sHB-EGF used in the following experiments was determined, we next performed cell migration and invasion assays. sHB-EGF treatment (0.125 μg/ml) dramatically increased SKOV3 cell migration as manifested by increased wound closure in the wound-healing assays (Fig. 2a, b). In addition, in transwell invasion assays, sHB-EGF treatment significantly increased the number of the cells which penetrated the matrigel and moved to the outer side of the transwells (Fig. 2c, d). Similar results were obtained when HO-8910 cells were treated with sHB-EGF (Fig. S3).

In order to further confirm the activities of sHB-EGF on cell migration and invasion, we used shRNA to silence the expression of HB-EGF (Fig. 2e) in SKOV3 cells. HB-EGF knockdown in SKOV3 decreased cell migration and invasion (Fig. 2f, g). These results suggested that sHB-EGF produced by SKOV3 cells can stimulate migration and invasion of the cells by autocrine and paracrine. These data demonstrated that sHB-EGF can significantly increase migration and invasion of SKOV3 and HO-8910 cells. Since metastasis is the major challenge for ovarian cancer treatment, we therefore focused on the effects of sHB-EGF on cell migration and invasion.

### Biopanning of sHB-EGF binding peptides

From the above data, we can conclude that sHB-EGF plays an important role in promoting migration and invasion of ovarian cancer cells. sHB-EGF is a promising target for ovarian cancer therapy.

**Fig. 3:**
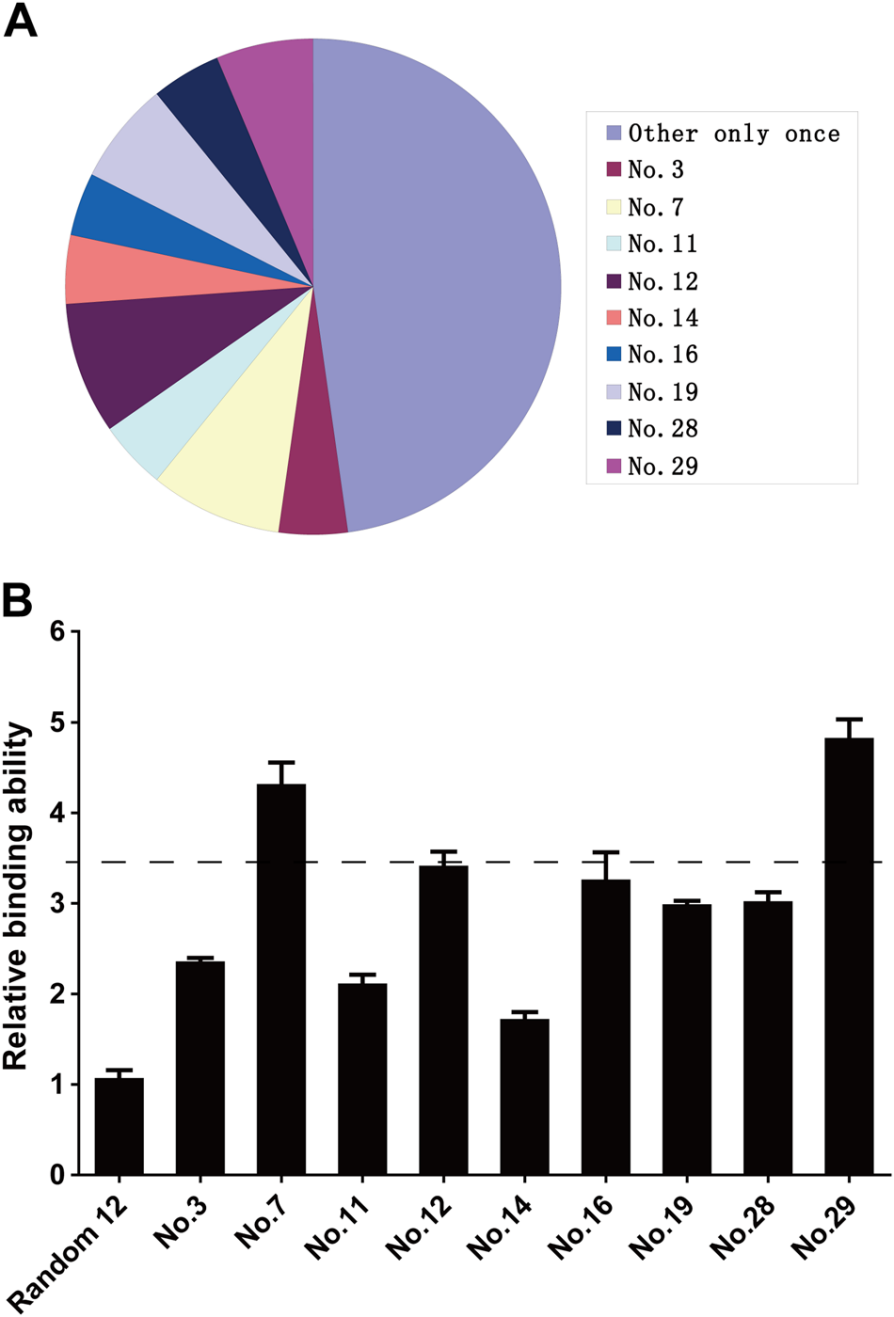
Selection of sHB-EGF binding peptides. **a** Results of biopanning of sHB-EGF binding peptides by phage display. Nine peptide sequences repeated for 2–4 times. **b** Quantification of binding abilities of peptides using enzyme-linked immunosorbent assay (ELISA). The relative binding abilities were calculated using the following equation: Absorbance when sHB-EGF coated/Absorbance when bovine serum albumin (BSA) coated. Mean ± SD, *n* = 3

In order to obtain the peptides which can interfere with the functions of sHB-EGF, novel peptides were searched using phage display. After four rounds of biopanning, 31 different peptide sequences from 46 isolated colonies were obtained. Nine peptides repeated for 2–4 times (Fig. 3a and Table 1) were selected as the candidates.

**Table 1:** Peptide sequenced in the fourth round

Serial number	Peptide sequence	Repeat times	Percentage
1	ALGDSCRYCRLL	1	2.17
2	ATEERSRIWMFL	1	2.17
3	AWADQPVTAPNR	2	4.35
4	CVASARGAQIGM	1	2.17
5	DDFRVWWPNFPR	1	2.17
6	DPVGLGGWWAKV	1	2.17
7	DRWVARDPASIF	4	8.7
8	DSSQWDKIYSWT	1	2.17
9	GFAVGARDSLMF	1	2.17
10	GSAPLLTVDTSK	1	2.17
11	HLTTTHPEPPYG	2	4.35
12	HPSALMKPTSHA	4	8.7
13	HSKAFPVLYPLR	1	2.17
14	IPLGRDGGSYQR	2	4.35
15	LGHSGGPTRPSW	1	2.17
16	MDENVATNQLMI	2	4.35
17	MLDQRPMSSYAG	1	2.17
18	MSLDSFRVDRRA	1	2.17
19	NPHAPSSFYEAY	3	6.52
20	QGMVAESYSPLS	1	2.17
21	SALKGLFPADHH	1	2.17
22	SLECDELIHSQI	1	2.17
23	STLGFNPPAILP	1	2.17
24	STPGCCAHDHFR	1	2.17
25	SVPMGSLASLES	1	2.17
26	TAHASLDDQGLR	1	2.17
27	TPQSFWQKGSLV	1	2.17
28	TSSPLTRWSSSL	2	4.35
29	TVGLPMTYYMHT	3	6.52
30	VSGQRSVGTPLS	1	2.17
31	WDFRQWWQPSGG	1	2.17
**Total**		**46**	**100.0**

To further narrow down the range of the candidates, we performed phage enzyme-linked immunosorbent assay (ELISA) to examine the binding abilities of the peptide candidates to sHB-EGF. As shown in Fig. 3b, most peptides showed a mild affinity to sHB-EGF. Therefore, we chose peptides no. 7 and no. 29 which have the highest affinities to sHB-EGF in our experiment to do the following study.

### Peptides no. 7 and no. 29 efficiently inhibited the function of sHB-EGF in promoting cell migration

**Fig. 4:**
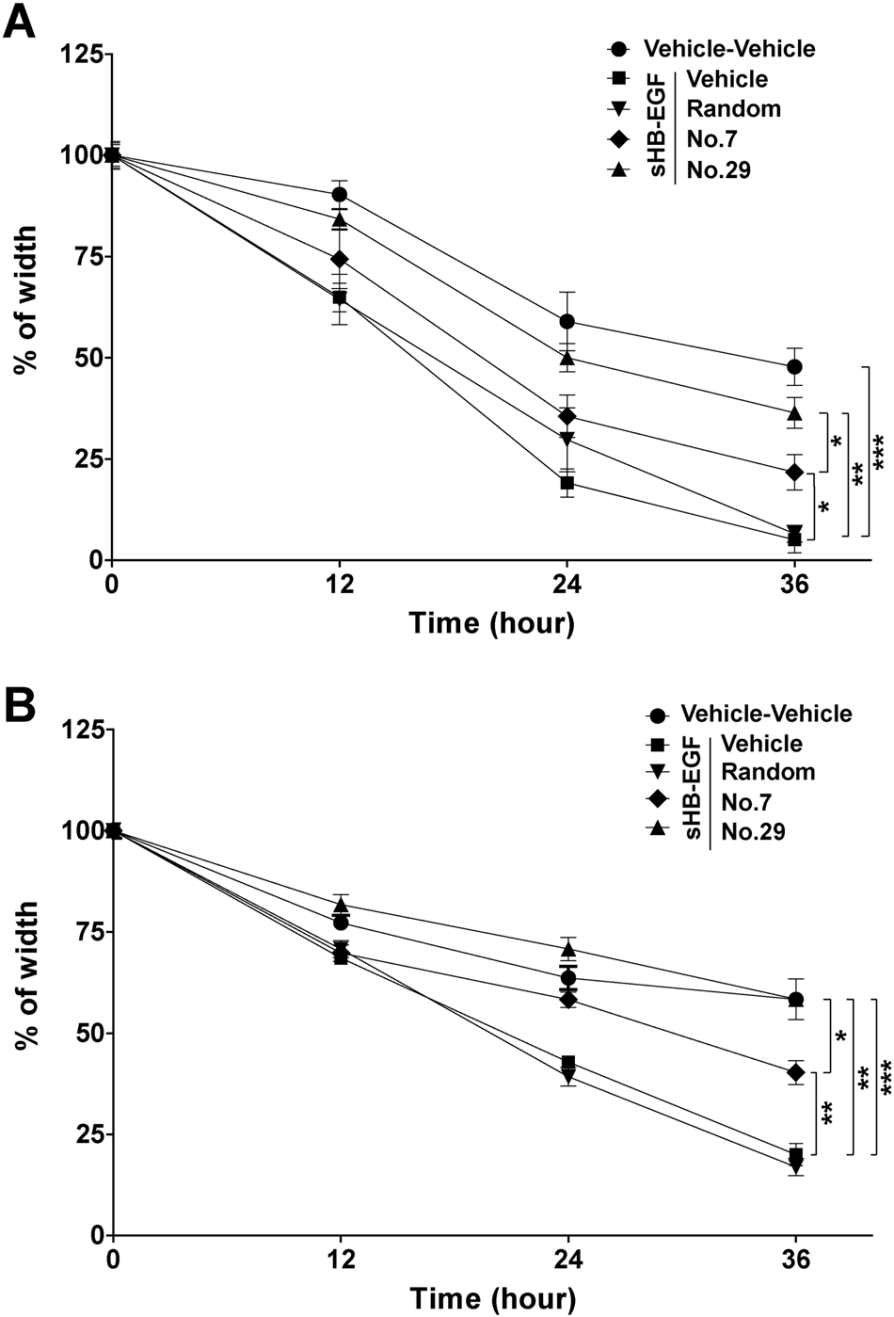
The effects of peptides no. 7 and no. 29 on cell migration promoted by sHB-EGF. **a** The statistical analysis of wound-healing assays on SKOV3 cells treated as indicated in the figure. **b** The statistical analysis of wound-healing assays on HO-8910 cells treated as indicated in the figure. Mean ± SD, *n* = 3. ^#^*P* > 0.05, *P < 0.05, **P < 0.01, ***P < 0.001

As the next step, we evaluated the biological activities of no. 7 and no. 29 peptides on cell migration promoted by sHB-EGF. In order to exclude the possibility of nonspecific effect in peptide treatment, a 12-AA peptide with a random sequence (Random) was synthesized and used as a negative control. Both no. 7 and no. 29 peptides were found to significantly inhibit sHB-EGF promoted migration of SKOV3 (Fig. 4a) and HO-8910 (Fig. 4b) cells. Interestingly, we observed that the inhibitory effect of peptide no. 29 was stronger than that of no. 7.

### Peptides no. 7 and no. 29 efficiently inhibited the function of sHB-EGF in promoting cell invasion

**Fig. 5:**
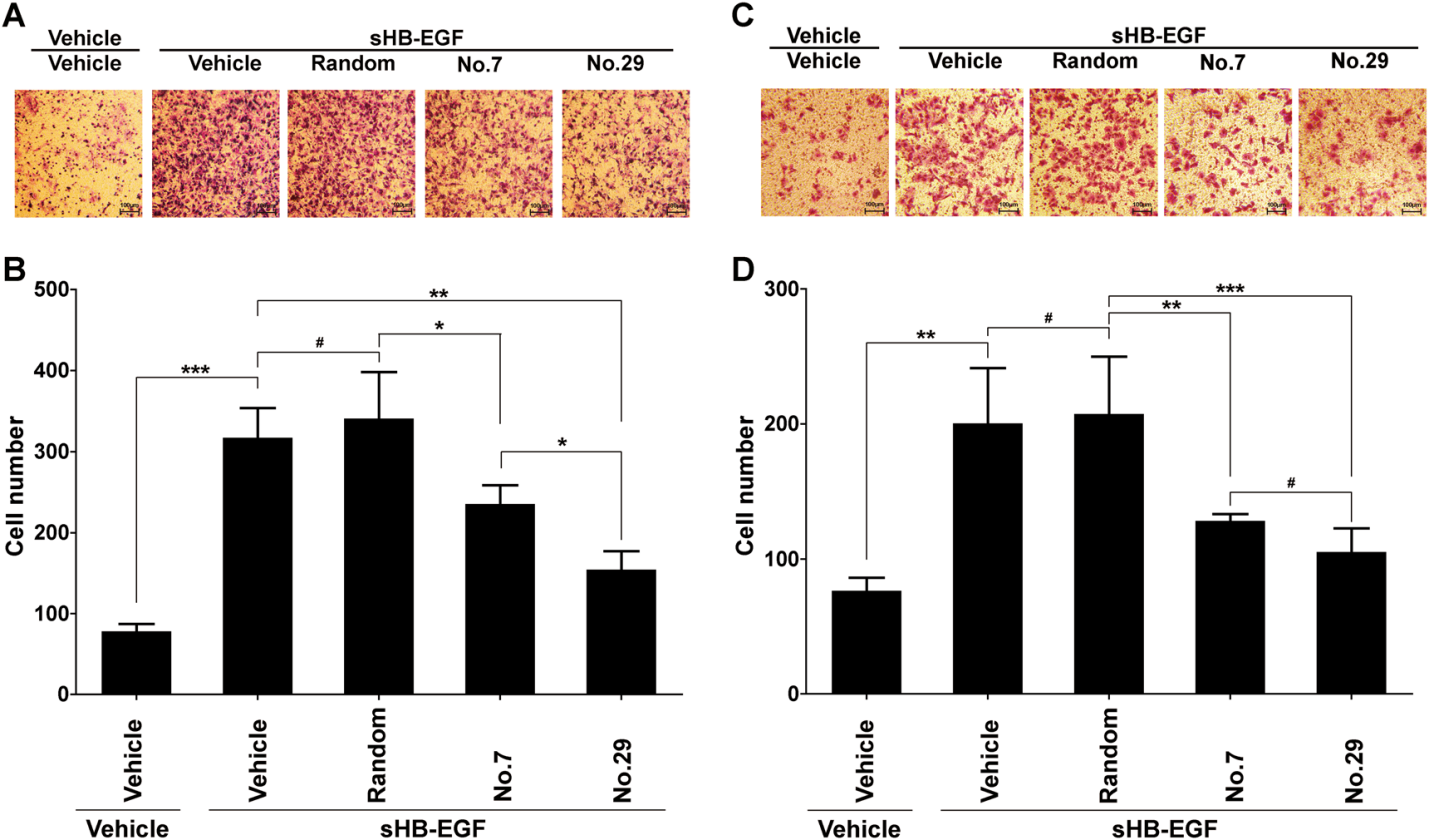
The effects of peptides no. 7 and no. 29 on cell invasion promoted by sHB-EGF. **a,** **b** The representative pictures and statistical analysis of transwell invasion assays on SKOV3 cells treated as indicated in the figure. **c,** **d** The representative pictures and statistical analysis of transwell invasion assays on HO-8910 cells treated as indicated in the figure. Mean ± SD, *n* = 3. ^#^*P* > 0.05, *P < 0.05, **P < 0.01, ***P < 0.001

After that, we assessed the biological activities of peptides no. 7 and no. 29 in cell invasion promoted by sHB-EGF. As expected, both peptides no. 7 and no. 29 suppressed increased cell invasion induced by sHB-EGF treatment in SKOV3 (Fig. 5a, b) and HO-8910 cells (Fig. 5c, d). Consistently, the inhibitory effect of peptide no. 29 is stronger than that of no. 7 in SKOV3 cells.

### Peptides no. 7 and no. 29 suppressed cell migration and invasion promoted by sHB-EGF through inhibition on the EGFR signaling pathway

**Fig. 6:**
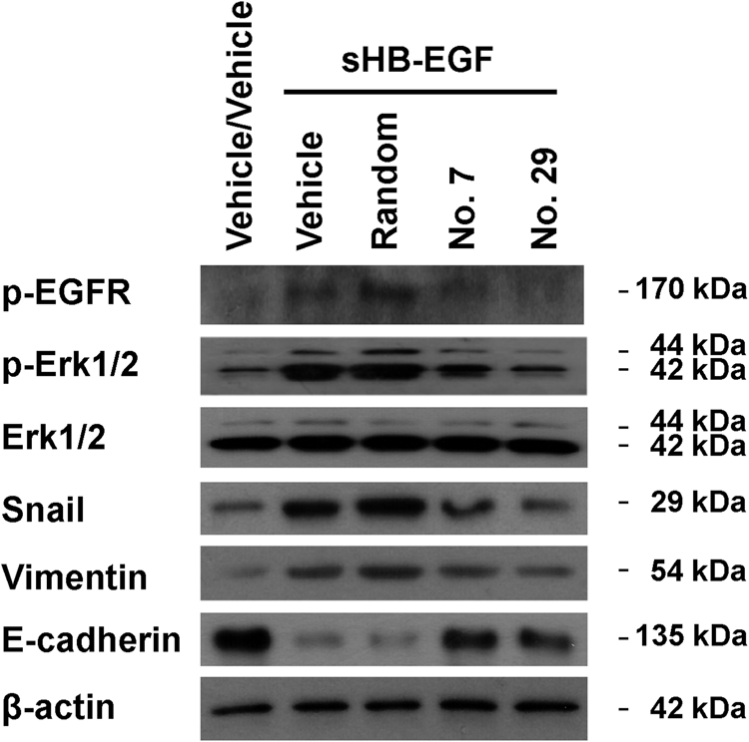
The effects of peptides no. 7 and no. 29 on EGFR activation induced by sHB-EGF treatment. The protein levels of p-EGFR, p-Erk1/2, total-Erk1/2, Snail, Vimentin, and E-cadherin in SKOV3 cells with the indicated treatments

After confirmed the biological activities of the candidate peptides, we sought to explore the underlying molecule mechanisms. It is well accepted that Snail, Vimentin, and E-cadherin play key roles in cell migration and invasion^23–26^. It has been reported that activation of the EGFR pathway can promote cell migration and invasion through upregulation of Snail^27–31^. In addition, Snail regulates Vimentin (positively) and E-cadherin (negatively) at the transcriptional level^32,33^. Consistent with a previous study^34^, in our experiment, sHB-EGF treatment activated the EGFR signaling pathway as manifested by increased levels of p-EGFR and p-ERK (Fig. 6). Treatment with peptide no. 7 or no. 29 efficiently attenuated activation of EGFR signaling stimulated by sHB-EGF. Consistently, sHB-EGF treatment increased protein levels of Snail and Vimentin and decreased that of E-cadherin. Treatment with peptide no. 7 or no. 29 inhibited the activities of sHB-EGF in regulating protein levels of Snail, Vimentin, and E-cadherin. These data strongly suggested that peptides no. 7 and no. 29 suppress cell migration and invasion by attenuating sHB-EGF-induced EGFR activation.

### The activities of peptides on tumor growth and migration in vivo

**Fig. 7:**
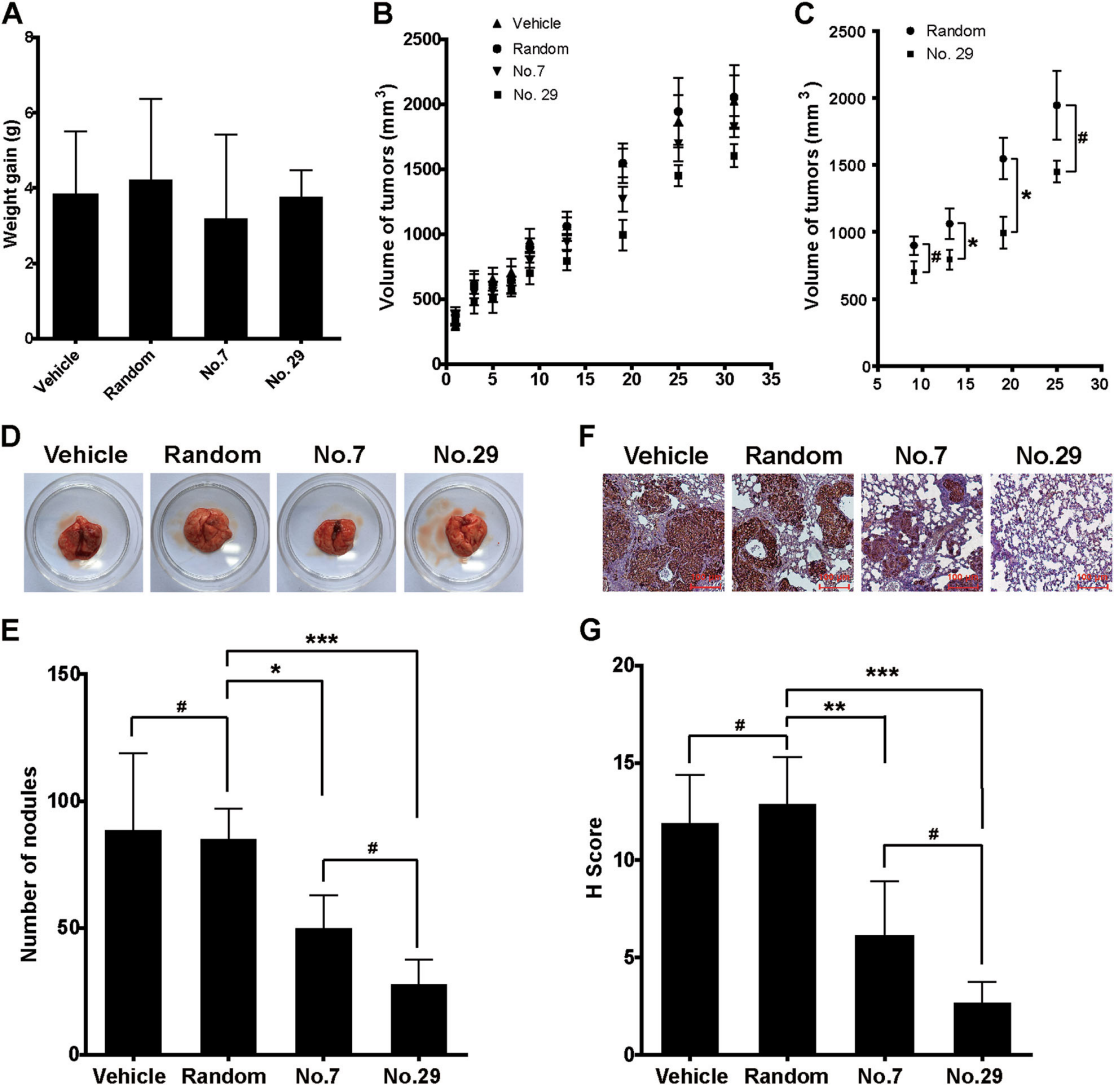
The in vivo activities of peptides no. 7 and no. 29. **a** Weight gain of mice during the experiment. **b,** **c** The growth curves of tumors in mice with different treatments. **d** The representative pictures of lungs in different groups. **e** Statistical analysis of nodules in lungs. **f** The representative pictures of immunohistochemistry (IHC) using anti-HLA antibody. **g** Statistical analysis of **f**. Mean ± SD, *n* = 5. ^#^*P* > 0.05, *P < 0.05, **P < 0.01, ***P < 0.001

After we confirmed the activities of peptides no. 7 and no. 29 in suppressing cell migration and invasion promoted by sHB-EGF in vitro, we started to test the effects of these peptides in tumor growth and migration in a xenograft mouse model. The results showed that none of the peptides had significant effects on weight gain (Fig. 7a) or tumor growth (Fig. 7b) at the end of the study. However, we indeed found that peptide no. 29 transiently inhibited tumor growth during the experiment (Fig. 7c). More importantly, peptides no.7 and no. 29 dramatically inhibited pulmonary migration of SKOV3 cells. As shown in Fig. 7d, e, the administration of peptides no. 7 and no. 29 significantly decreased the numbers of nodules in the lungs of the mice. Consistent with this, treatment using peptide no. 7 or no. 29 dramatically decreased the H-scores in immunohistochemistry (IHC) assay using anti-HLA-A antibody which specifically identifies human SKVO3 cells in a mouse lung (Fig. 7f, g). These data demonstrated that peptides no. 7 and no. 29 can efficiently inhibited migration and invasion of SKOV3 cells in mice.

## Discussion

The piling evidence has indicated HB-EGF as an important target in combating tumorigenesis and metastasis of ovarian cancer^35^. Peptide-based therapy has become a hot spot for drug development. In this study, several sHB-EGF binding peptides were obtained using phage display. Our data showed that peptides no.7 and no. 29 can significantly suppress the activities of sHB-EGF in promoting migration and invasion of SKOV3 and HO-8910 cells. It is widely accepted that activation of the EGFR-Erk pathway can upregulate the expression of Snail, a key role molecular in regulation of cell migration and invasion^36–39^. Our mechanism study showed that peptides no. 7 and no. 29 inhibited sHB-EGF promoted cell migration and invasion by attenuating sHB-EGF-induced EGFR activation. More importantly, our results revealed that peptides no. 7 and no. 29 can significantly suppress migration of SKOV3 cells in the mouse model.

In our phage display experiment, we could not obtain a dominant peptide sequence. Because sHB-EGF (only 87 AA) is relatively short, we reason that it is difficult for sHB-EGF to form ‘deep pits’ to offer good opportunities for peptides to bind tightly. Therefore, only peptides with mild affinities and limited repeated times were obtained in the sequenced pool. Consistent with this notion, ELISA results showed that the relative binding abilities of the peptides to sHB-EGF were in the low to mild range (1.7–4.8). More excitingly, we found that two of our peptide candidates, no.7 and no. 29, can inhibit the activities of sHB-EGF in promoting migration and invasion of ovarian cancer cells in vitro and in vivo. It seems the inhibitory activity of peptide no. 29 is stronger than that of no. 7. Due to the technical limitation, we could not identify the specific binding sites between sHB-EGF and the peptide candidates, but the results suggested that the binding sites are quite influential on peptide’s ability to exert inhibitory effects on sHB-EGF.

For the in vivo experiment, we did not apply sHB-EGF treatment on mice; however, peptides no. 7 and no. 29 still showed strong activities in inhibiting migration of SKOV3 cells. We reason here that there is plenty of sHB-EGF production in mice^40,41^, which stimulates migration and invasion of SKOV3 cells. Peptides no. 7 and no. 29 inhibited the functions of endogenous sHB-EGF, therefore suppressed migration and invasion of SKOV3 cells.

We have obtained the promising peptides which showed potent activities in blocking functions of sHB-EGF, in the near future, we will endeavor to optimize the sequences and structures of peptides for better activities and stabilities in vivo for ovarian cancer therapy.

## Materials and methods

### Cell culture

Ovarian cancer cell lines, SKOV3 and HO-8910, were purchased from American Type Culture Collection and maintained in RPMI 1640 with 1% penicillin G, 1% streptomycin, and 10% fetal bovine serum (FBS). Cells were cultured in a humidified CO_2_ incubator at 37 °C.

### Bacterial strains, plasmids, and antibodies

*Escherichia coli* strain BL21 (DE3) was used as host cells in sHB-EGF expression. pET-30a was obtained from Novagen. pLKO.1, pLP1, pLP2, and pLPSVG were purchased from OpenBiosystems. pIRES-proHB-EGF was obtained from Addgene. Ph.D.-12 and enterokinase were purchased from New England Lab. Ni-NTA was purchased from Qiagen. Heparin-conjugated agarose was purchased from GE Healthcare. Anti-His-tag antibody was from Proteintech. Antihuman HB-EGF antibody was from R&D systems. Anti-HLA-A (major histocompatibility complex, class I, A) antibody was from Abcam. Antibodies targeting p-EGFR, ERK1/2, and p-ERK1/2 were purchased from Ruiying Biological. Antibodies to Vermentin, Snail, and E-cadherin were purchased from Santa Cruz. Other chemicals are at least analytic grade if without other specification.

### Plasmid construction

The DNA fragment coding human sHB-EGF was amplified from pIRES-proHB-EGF using PCR reaction with the following primers: 5′-CGGGATCCGACTTGCAAGAGGCAGAT-3′ and 5′-CCAAGCTTTCATGGGAGGCTCAGCCC-3′. The DNA fragment was inserted into pET-30a with the BamHI and Hind III sites to construct pET-30a-His-sHB-EGF. To construct the HB-EGF short hairpin RNA (shHB-EGF), we synthesized the following oligonucleotides: 5′-CCGGTGGAGAATGCAAATATGTGCTCGAGCACATATTTGCATTCTCCATTTTT-3′ and 5′-AATTAAAAATGGAGAATGCAAATATGTGCTCGAGCACATATTTGCATTCTCCA-3′. After annealing, the fragments were ligated into the pLKO.1 vector between AgeI and EcoRI sites.

### Production and administration of pseudovirus

ShHB-EGF or pLKO.1 was transfected into 293T cells together with pLP1, pLP2, and pLPSVG using Lipofectamine 2000 reagent. Medium containing pseudovirus was collected 72 h after transfection and applied on the target cells with appropriate dilution for 8 h. Cells were selected with 1 μg/ml puromycin to enrich the transducted cells.

### Expression and purification of sHB-EGF

Plasmid pET-30a-sHB-EGF was transformed into BL21 (DE3). When OD value of the bacterial culture reached 0.6, IPTG was added to 0.8 mM. The bacteria were cultured at 25 °C for another 12 h. We initially purified (His)_6_-sHB-EGF by affinity chromatography using Ni-NTA and heparin-conjugated agarose. In order to remove the tag, 0.001% enterokinase was used and then the protein was purified through heparin-conjugated agarose again. Purified sHB-EGF was freeze-dried and stored at −80 °C for other assays.

### High performance liquid chromatography (HPLC)

Purified sHB-EGF (30  μg) was freeze-dried and dissolved in 150 μl of dissolving buffer (water plus 0.1% trifluoroacetic acid). For each injection, 50 μl of the sample was used, and the volume of the sample loop is 20 μl. As for column, Agilent ZORBAX 300SB-C8 was chosen. The mobile phase A was acetonitrile plus 0.1% trifluoroacetic acid, and the phase B was water plus 0.1% trifluoroacetic acid. The elution gradient was set as: 0 min, 10% A plus 90% B; 30 min, 100% A. The flow rate was set at 1 ml/min and the column temperature was set at 30 °C. The detection wavelength was 280 nm. The retention times and the peak areas were analyzed with the software supplied by the manufacturer.

### Western blotting

The protein was separated on a 15% SDS-PAGE gel and then transferred to a PVDF membrane. After blocking (5% skim milk in Tris-NaCl buffer (TBS), 25 mM Tris, 0.15M NaCl, pH 7.3) for 1 h at room temperature, the membrane was washed three times for 5 min with TBS containing 0.05% tween. Then the diluted primary antibody was added into TBS and incubated with the membrane overnight at 4 °C. The membrane was washed and incubated with an HRP-labeled secondary antibody (Cell Signaling) for 1 h. The membrane was developed using the ECL chemiluminescence’s system (Thermo Scientific).

### Phage display

Six hundred microliters of sHB-EGF (100 μg/ml in NaHCO_3_, pH 8.6) was coated on polystyrene 6-well cell culture plate overnight at 4 °C. Other steps were performed according to manual of Ph.D-12. In order to avoid nonspecific binding, the phage elusion in each round was incubated with a new plate for 20 min at room temperature. After 4 rounds of biopanning, the isolated colonies of *E. coli* ER2738 were cultured and the remained phages were sequenced.

### Phage ELISA

Two hundred microliters of sHB-EGF (100 μg/ml in NaHCO_3_, pH 8.6) or bovine serum albumin (BSA) at the same concentration was coated on polystyrene 96-well plate. After overnight incubation at 4 °C, plates were washed with TBST and blocked with 5 mg/ml BSA in NaHCO_3_. Candidate phages with a titer around 10^10^ were added to each coated well, and the plate was incubated for 1 h at room temperature. After washed by 0.5% TBST for three times, the binding phages were detected by a HRP-conjugated mouse anti-M13 monoclonal antibody (GE Healthcare). Absorbance was measured at 405 nm. The relative absorbance was calculated using the following equation:

Absorbance when sHB-EGF coated/Absorbance when BSA coated

### Cell proliferation assay

Cells (4 × 10^3^/well) were seeded on a 96-well plate and cultured with at 37 °C. After 24 h, sHB-EGF was added to reach various concentrations ranging from 0.002 to 0.25 μg/ml. After 72 h, 10 μl of MTT (5 mg/ml) was added to each well and the cells were incubated at 37 °C for ~1.5 h. Hundred microliters of Dimethyl sulfoxide (DMSO) was added to each well to dissolve violet crystal. Finally, absorbance at 550 nm was measured using microplate reader.

### Wound-healing assay

Cells were grown to 90% confluence in a six-well plate and serum-starved for 24 h, and then a wound was made on the monolayer of cells by using a sterile 100 μl pipette tip. Cells were washed with PBS and cultured in the designated media. The wound was photographed under microscope at different times. sHB-EGF was added to reach the concentration of 0.125 μg/ml. For peptide treatment, a peptide (a stock solution was made by dissolving a peptide in DMSO at 5000 μM) was added to reach a final concentration of 25 μM.

### Transwell invasion assay

Cell migration was assessed using a 24-well transwell tissue culture plate with inserts covered with 8-μm pore size membrane. Matrigel was placed in the up chamber incubated at 37 °C for 12 h. Cells (6 × 10^4^ cells/well) in 200 μl of serum-free medium were added into the up chamber and 500 μl of medium containing 10% FBS was added to the bottom chamber. After 36 h, the cells that had passed through gel to the bottom of the inserts were stained with crystal violet, photographed and counted. sHB-EGF was added to reach 0.125 μg/ml in the up chamber. For peptide treatment, a peptide was added to reach the concentration of 25 μM.

### Administration of peptides on xenograft mouse model

The activities of peptides no. 7 and no. 29 were evaluated in a mouse xenograft model. All animal experiments were performed in compliance with the guidelines of Jiangsu University for animal care and administration. SKOV3 cells (1 × 10^6^) were injected into the flank of a nude mouse (6-week-old). When the average volume of the tumors reached 100 mm^3^, the mouse was randomly divided into four groups, namely, Vehicle, Random, no.7, and no. 29 (5 for each group). For Random, no. 7 or no. 29 group, peptide was dissolved in a saline solution and injected (10 mg/kg) i.v. every 2 days for 4 weeks. The Vehicle group was injected with a saline solution. The weight and tumor volumes of mice were measured throughout the whole experiment. At the end of the experiment, the mice were executed, the tumors were weighed and lungs of mice were isolated. After a brief wash with PBS, tumor nodules on each lung were counted through visual inspection. After that, lungs were fixed and subjected to IHC staining using anti-HLA-A antibody (an antibody specifically identifying human major histocompatibility complex). H-score was assessed by two pathologists independently.

### Statistics

Results were expressed as the mean ± S.D. Data were analyzed by Student’s *t*-test for comparison between two groups and one-way ANOVA when the data involved three or more groups. A value of *P* < 0.05 was considered statistically significant.

## Supplementary information

Supplementary figure legends

Supplementary Figure 1

Supplementary Figure 2

Supplementary Figure 3
