# Supplementary material for: Discovery of HB-EGF binding peptides and their functional characterization in ovarian cancer cell lines
Source: Cell Death Discov. 2019 Mar 25;5:82. doi: 10.1038/s41420-019-0163-9 (PMC6433920; doi:10.1038/s41420-019-0163-9)
Supplement: Supplementary file 1 — Supplementary figure legends [file 41420_2019_163_MOESM1_ESM.doc]

**Supplementary figure legends**

**Figure S1. High Performance Liquid Chromatography analysis of purified sHB-EGF.** Protein was detected by UV absorbance at 280 nm. The retention time was used to identify the peaks. The experiments were repeated for 3 times and similar results were obtained. The major peak (retention time = 17.318 min) accounted for 91.2 (±0.65) % of the total peak area, Mean±SD, n=3.

**Figure S2. The effects of sHB-EGF on growth of SKOV3 and HO-8910 cells.** A and B, the relative viabilities of SKOV3 and HO-8910 cells treated with sHB-EGF at different concentrations for 72 hours. The viability of cells was measured using MTT assay, Mean±SD, n=3, *, P<0.05; **, P<0.01.

**Figure S3.** **sHB-EGF treatment significantly promoted migration and invasion of HO-8910 cells.** A, statistical analysis of wound healing assays; for sHB-EGF treatment, sHB-EGF was added to 0.125 μg/ml. B, statistical analysis of transwell invasion assay. Mean±SD, n=3. **, P<0.01; ***, P<0.001; compared with the control group.
