# Supplementary figures and images for: Discovery of HB-EGF binding peptides and their functional characterization in ovarian cancer cell lines
Source: Cell Death Discov. 2019 Mar 25;5:82. doi: 10.1038/s41420-019-0163-9 (PMC6433920; doi:10.1038/s41420-019-0163-9)

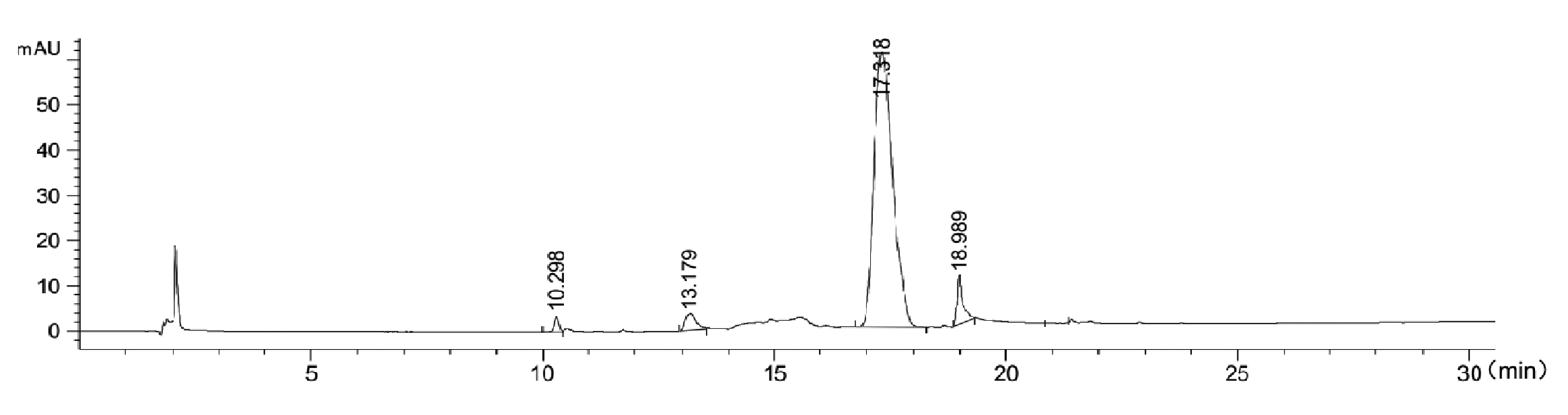

Supplement: Supplementary file 2 — Supplementary Figure 1 [file 41420_2019_163_MOESM2_ESM.tif]

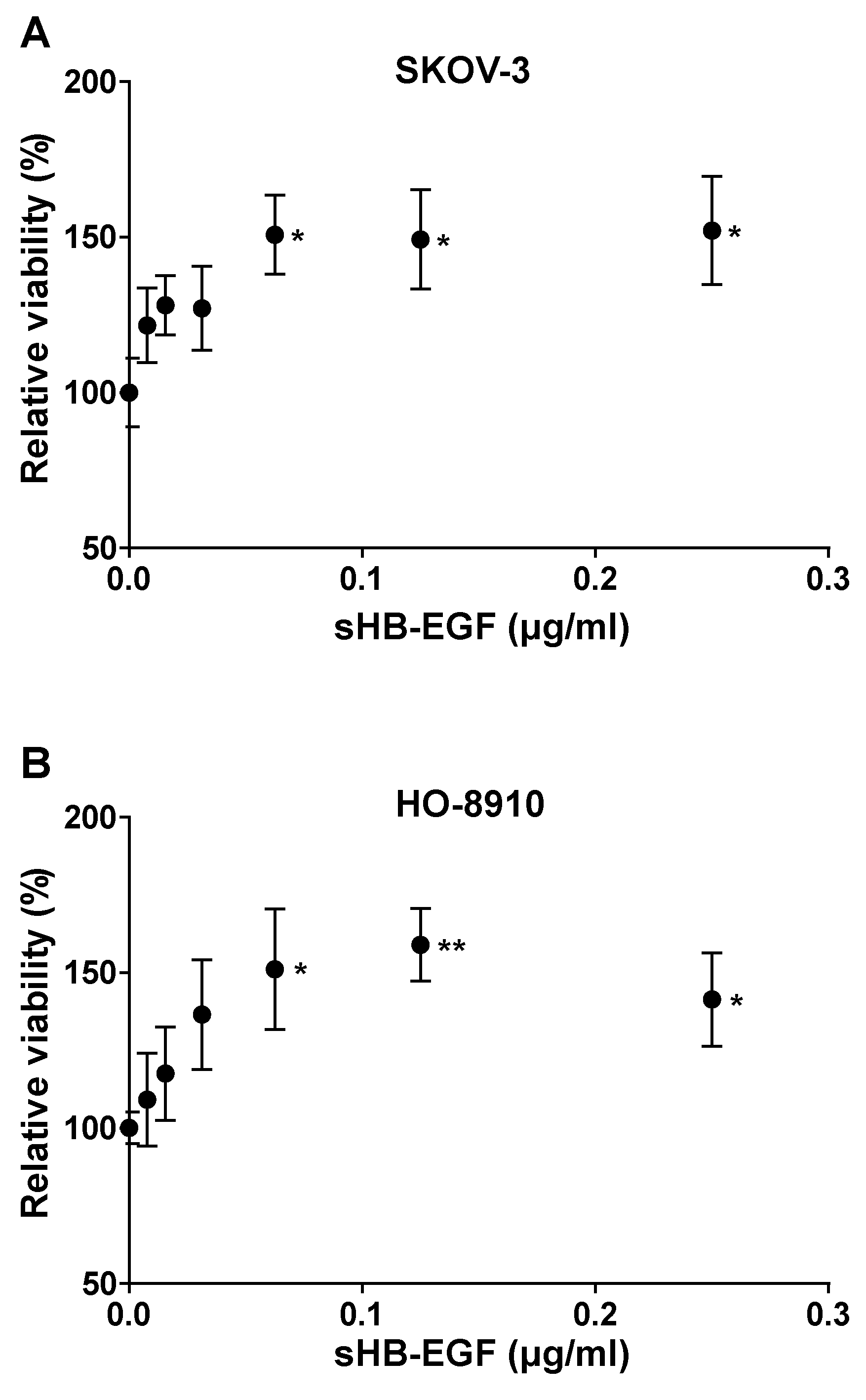

Supplement: Supplementary file 3 — Supplementary Figure 2 [file 41420_2019_163_MOESM3_ESM.tif]

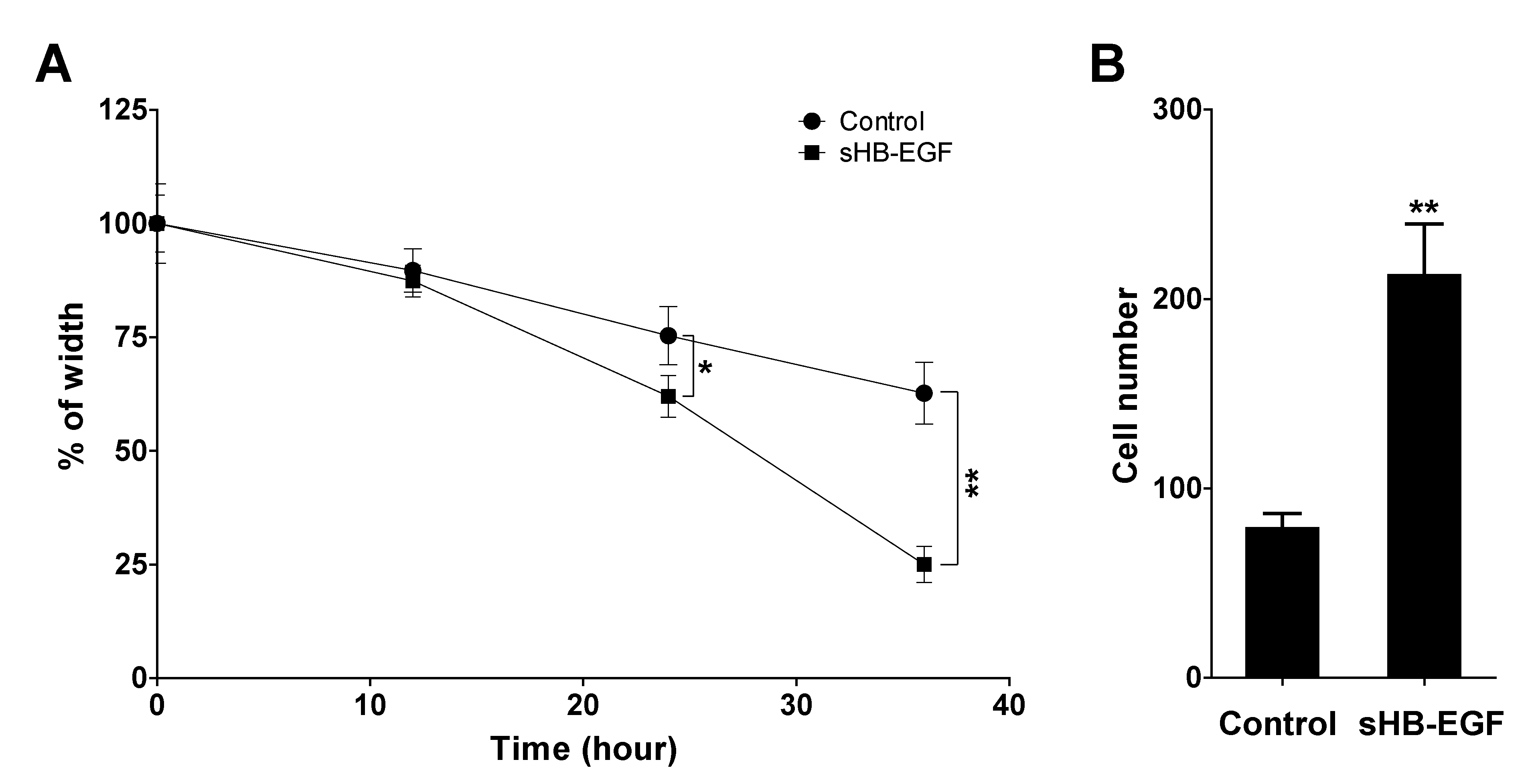

Supplement: Supplementary file 4 — Supplementary Figure 3 [file 41420_2019_163_MOESM4_ESM.tif]
